# Single-cell atlas of dental pulp stem cells exposed to the oral bacteria *Porphyromonas gingivalis* and *Enterococcus faecalis*

**DOI:** 10.3389/fcell.2023.1166934

**Published:** 2023-05-23

**Authors:** Wen Zhang, Tiansong Xu, Xueying Li, Yifei Zhang, Xiaoying Zou, Feng Chen, Lin Yue

**Affiliations:** ^1^ Department Cariology, Endodontology and Operative Dentistry, Peking University School and Hospital of Stomatology & National Center for Stomatology & National Clinical Research Center for Oral Diseases & National Engineering Research Center of Oral Biomaterials and Digital Medical Devices, Beijing, China; ^2^ Central Laboratory, Peking University School and Hospital of Stomatology, & National Center for Stomatology & National Clinical Research Center for Oral Diseases & National Engineering Research Center of Oral Biomaterials and Digital Medical Devices, Beijing, China; ^3^ Center of Stomatology, Peking University Hospital, Beijing, China

**Keywords:** regenerative endodontic treatment, human dental pulp stem cell, single-cell RNA sequencing, Porphyromonas gingivalis, *Enterococcus faecalis*, cell-cell interactions and development

## Abstract

**Introduction:** *Porphyromonas gingivalis* and *Enterococcus faecalis* promote the development of pulpitis and periapical periodontitis. These bacteria are difficult to eliminate from the root canal systems, leading to persistent infection and poor treatment outcomes. We explored the response of human dental pulp stem cells (hDPSCs) to bacterial invasion and the mechanisms underlying the impact of residual bacteria on dental pulp regeneration.

**Methods:** Single-cell sequencing was used to categorize the hDPSCs into clusters based on their response to *P. gingivalis* and *E. faecalis*. We depicted a single-cell transcriptome atlas of hDPSCs stimulated by *P. gingivalis* or *E. faecalis*.

**Results:** The most differentially expressed genes in the Pg samples were *THBS1*, *COL1A2*, *CRIM1*, and *STC1*, which are related to matrix formation and mineralization, and *HILPDA* and *PLIN2*, which are related to the cellular response to hypoxia. A cell cluster characterized by high expression levels of *THBS1* and *PTGS2* was increased after *P. gingivalis* stimulation. Further signaling pathway analysis showed that hDPSCs prevented *P. gingivalis* infection by regulating the TGF-β/SMAD, NF-κB, and MAPK/ERK signaling pathways. Differentiation potency and pseudotime trajectory analyses showed that hDPSCs infected by *P. gingivalis* undergo multidirectional differentiation, particularly to the mineralization-related cell lineage. Furthermore, *P. gingivalis* can create a hypoxia environment to effect cell differentiation. The Ef samples were characterized by the expression of *CCL2*, which is related to leukocyte chemotaxis, and *ACTA2*, which is related to actin. There was an increased proportion of a cell cluster that was similar to myofibroblasts and exhibited significant *ACTA2* expression. The presence of *E. faecalis* promoted the differentiation of hDPSCs into fibroblast-like cells, which highlights the role of fibroblast-like cells and myofibroblasts in tissue repair.

**Discussion:** hDPSCs do not maintain their stem cell status in the presence of *P. gingivalis* and *E. faecalis*. They differentiate into mineralization-related cells in the presence of *P. gingivalis* and into fibroblast-like cells in the presence of *E. faecalis*. We identified the mechanism underlying the infection of hDPSCs by *P. gingivalis* and *E. faecalis*. Our results will improve understanding of the pathogenesis of pulpitis and periapical periodontitis. Furthermore, the presence of residual bacteria can have adverse effects on the outcomes of regenerative endodontic treatment.

## 1 Introduction

Dental pulpitis and periapical periodontitis are common infections of the oral cavity (Tibúrcio-Machado et al., 2021; León-López et al., 2022). Several bacteria can participate these diseases, including those from the phyla Firmicutes, Actinomycetes, Fusobacteria, Spirochaetes, and *Bacteroides* (Siqueira & Roca., 2022). Culture and identification techniques have shown that Firmicutes and *Bacteroides* are closely associated with the development of irreversible pulpitis and periapical periodontitis (Rôças et al., 2016; Sánchez-Sanhueza et al., 2018; de Brito et al., 2020; Zahran et al., 2021; Buonavoglia et al., 2023).

*Enterococcus faecalis* is a Gram-positive facultative anaerobe from the Firmicutes phylum. This bacterium is highly resilient (Love, 2001) and typically infects the dental pulp, leading to persistent root canal infection (Hancock et al., 2001; Kayaoglu AND Orstavik, 2004; Stuart et al., 2006; Zhu et al., 2010). The effects of *Enterococcus faecalis* on the pulp and periapical tissue are unclear. The bacteria releases byproducts such as lysase, gelatinase, hyaluronidase, and cytolysin, which cause tissue damage (Kayaoglu AND Orstavik, 2004) or modulate the immune responses of pulp cells, leading to further tissue damage (Sipert et al., 2010; Fransson et al., 2014). Furthermore, this bacteria inhibits osteoblast differentiation (Karygianni et al., 2012; Park et al., 2015) and upregulates the expression of osteogenic genes (*RUNX2*, *ALP*, *COL1A1*, and *ALP*) in human dental pulp stem cells (hDPSCs) (Lee et al., 2022), which might affect the repair of the pulp and periapical lesions. However, the response of hDPSCs to *E. faecalis* is incompletely understood.

*Porphyromonas gingivalis* is a Gram-negative anaerobe from the *Bacteroides* phylum and is the primary bacterial pathogen involved in periodontal disease. This microbe invades the dental pulp through the channels within the periodontal pocket, leading to retrograde pulpitis. *P. gingivalis* has been detected in the pulp and adjacent deep periodontal pocket of infected patients (Li et al., 2014), and is typically found in the apical region of the root canal. It is a dominant bacteria found in persistent periapical lesions (Zakaria et al., 2015). *P. gingivalis* and its byproduct, lipopolysaccharide (LPS), can affect dental pulp cells (Yang et al., 2003; Sipert et al., 2013; Biedermann et al., 2014; Liu et al., 2015). When hDPSCs are exposed to the LPS produced by *P. gingivalis* (Pg-LPS), the expression of genes involved in mineralization (*DSPP* and *OCD*) is downregulated (Yamagishi et al., 2011). Although *P. gingivalis* facilitates immune evasion in periodontal tissue (Zheng et al., 2021), its role in dental pulp tissue needs further investigation.

In addition to causing infections, bacteria negatively impact treatment outcomes. Regenerative endodontic treatment (RET) involves biological processes that aim to replace damaged tooth structures and cells in the pulp-dentine complex (Murray et al., 2007; Hargreaves et al., 2013). However, histological studies have shown that the new tissue that forms in the canal space after pulp RET is more like bone, cementum, and periodontal ligament than like pulp or dentin (Altaii et al., 2017). Residual bacteria may negatively impact the outcomes of RET (Siqueira & Rocas., 2008; Verma et al., 2017; Zaky et al., 2020). The current root canal disinfection methods cannot completely remove bacteria, their biofilms and byproducts. Active *E. faecalis* and its metabolic products have been detected in the apical region after disinfection (Baca et al., 2011; Pinheiro et al., 2015; Zhang et al., 2015; Vishwanat et al., 2017). The presence of residual bacteria in root canals can affect the biological properties of endogenous and exogenous stem cells during pulp regeneration treatment, as well as the regenerated tissues (Fouad, 2011; Nosrat et al., 2015; Fouad, 2017). A previous study showed that the quantity of dentin-associated mineralized tissue was significantly less in teeth with vs. without residual bacteria (*E. faecalis*) (Siqueira & Rocas., 2008; Verma et al., 2017). Furthermore, Pg-LPS inhibits odontoblast differentiation (Yamagishi et al., 2011; Vishwanat et al., 2017), which inhibits pulp regeneration. However, the mechanisms underlying these effects are unclear.

Some studies have investigated the impact of two common bacteria, *E. faecalis* and *P. gingivalis*, on dental pulp tissue. These bacteria interact with cells during invasion of dental pulp tissue through various mechanisms, such as adhesion and internalization (Biedermann et al., 2014). Furthermore, bacteria possess multiple virulence factors, such as lysase, gelatinase, hyaluronidase, and cytolysin. A single virulence factor (Sipert et al., 2013) and related cytokines and genes alone will not reflect the overall cell response (Yamagishi et al., 2011; Lee et al., 2022). *In vitro* coculture models are widely used to explore the interactions among microorganisms and a host. This approach can be used to evaluate bacterial adhesion and invasion, and cellular immune and inflammatory reactions (Puschhof et al., 2021). Coculture models of the interactions between dental stem cells and *P. gingivalis* have demonstrated the occurrence of cell adhesion and internalization (Biedermann et al., 2014). Such models comprehensively reflect the interaction between cells and microorganisms.

We used single-cell sequencing to create a hDPSCs atlas following infection with *E. faecalis* or *P. gingivalis*. We analyzed the differentiation trends and pathway enrichment of clusters of infected and noninfected hDPSCs, constructed an interaction network of multiple cells, proposed their possible evolutionary trajectories, and explored the effects of bacterial invasion on hDPSCs differentiation and proliferation. Based on our results, we proposed some mechanisms underlying the impact of residual bacteria in root canal systems on dental pulp regeneration.

## 2 Materials and methods

### 2.1 Cell isolation and culture

hDPSCs were isolated as described previously (Liu et al., 2015). Impacted third molars were obtained from healthy individuals (*n* = 6; age: 18–23 years) at the Oral and Maxillofacial Surgery Department. The study protocol was approved by the Ethics Committee of Peking University School and Hospital of Stomatology (PKUSSIRB-202163047). Immediately after extraction, pulp tissues were minced into small pieces (0.1 × 0.1 × 0.1 cm^3^) and digested with 3 mg/mL collagenase type I (Worthington Biochemical Corp., Lakewood, NJ, United States) for 30 min at 37°C. The digested tissue and single cells were seeded into six-well plates with minimal essential medium-α (Gibco, Grand Island, NY, United States) supplemented with 10% fetal bovine serum (Kang Yuan Biology) and 1% penicillin-streptomycin (Gibco) under 5% CO_2_ at 37°C. The resulting heterogeneous populations of adherent, clonogenic dental stem/progenitor cells were analyzed for their cell surface marker expression by flow cytometry (positive for STRO-1, CD146, CD90, and CD105, and negative for CD45). The cells used in the present study were from passage 4 or 5.

### 2.2 Microbial strains and growth conditions

*E. faecalis* ATCC 29212 and *P. gingivalis* ATCC 33277 were gifted by the Central Laboratory of Peking University School of Stomatology. *E. faecalis* were grown aerobically (5% CO_2_, 37°C) in brain heart infusion medium, whereas *P. gingivalis* were grown anaerobically in brain heart infusion medium supplemented with 1% hemin and 0.5% vitamin K.

### 2.3 Coculture of hDPSCs and bacteria

For coculture, hDPSCs from six donors were mixed to reduce the impact of individual differences on the results. The cells were seeded at a density of 5 × 10^4^ cells/well in a 24-well plate. The final concentration of the bacterial suspension was measured based on the optical density at 630 nm. The microbial concentration was measured at optical density (630 nm) = 0.1 as 1 × 10^9^ CFU/mL, and the solution was diluted to achieve the desired multiplicity of infection. After cell adhesion, the cells were infected with live bacteria at a multiplicity of infection of 1:1 and incubated for 4 h at 5% CO_2_ and 37°C. Before harvesting, cells were incubated with 200 µM 4sU for 2 h in each group. 4sU is incorporated into new RNA during transcription and converted to a cytosine analogue using iodoacetamide (IAA) before RNA sequencing (Eramo et al., 2018). We used 4sU as a metabolic marker and quantified the newly synthesized RNA by labeling.

### 2.4 Single-cell RNA sequencing (scRNA-seq) using the singleron GEXSCOPE^®^ platform

The quality and concentration of the single-cell preparations were evaluated using a hemocytometer and an inverted microscope. Single-cell suspensions were prepared in phosphate-buffered saline (HyClone, Logan, UT, United States) at a concentration of 1 × 10^5^ cells/mL and loaded onto microfluidic devices. ScRNA-seq libraries were constructed using GEXSCOPE^®^ Single-Cell RNA Library Kit (Singleron Biotechnologies, Nanjing, China) according to the manufacturer’s protocol. Individual libraries were diluted to 4 nM and pooled for sequencing using Illumina novaseq 6,000 with 150 bp paired end reads.

### 2.5 Data alignment

Raw reads were processed to generate gene expression matrixes using CeleScope (https://github.com/singleron-RD/CeleScope) v1.9.0 pipeline. Briefly, low-quality reads were removed using CeleScope; then Cutadapt v1.17 (Martin, 2011) was used to trim the poly-A tail and adapter sequences. The cell barcode and unique molecular identifiers were extracted. Thereafter, we used STAR v2.6.1a (Dobin et al., 2013) to map the reads to the reference genome GRCh38 (ensembl version 92 annotation). The unique molecular identifier and gene counts of each cell were acquired using featureCounts v2.0.1 (Liao et al., 2014), and were used to generate expression matrix files, including total, new, and old RNA matrices, for subsequent analysis.

### 2.6 Data import, transformation and integration

We input the filtered feature barcode matrices using the function “Read10X” and “CreateSeuratObject.” Quality control, normalization, and downstream analysis were performed using the R package Seurat (ver. 4.0.2; https://github.com/satijalab/seurat). We extracted cells that expressed 200–4,000 genes and had < 15% mitochondrial genes to exclude low-quality cells and those with artifacts. Data were normalized to transform the gene expression matrices. The integrated data and correct batch of each group were analyzed by integrating data using “FindIntegrationAnchors” and “IntegrateData.”

### 2.7 Dimensionality reduction, cell clustering and identification

Sequenced cells were assigned a cell cycle phase “score” and scaled to reduce cell cycle heterogeneity. The first 50 PCs and the parameter resolution to 1 were used to cluster data into cell types. Cluster-specific genes were identified using “FindAllMarkers,” and cell clusters were manually annotated based on these genes.

### 2.8 Pathway analysis

Gene set over-representation of significantly upregulated cluster-defining genes was analyzed using the gsfisher R package (ver. 0.2; https://github.com/sansomlab/gsfisher/) and the corresponding gene sets were obtained from the gene ontology database.

### 2.9 Differentiation potency analysis

The differentiation potency score was calculated using the “AddModuleScore” function. The gene sets were used to rank the cells in the osteogenic, chondrogenic, adipogenic, endothelial, neurogenic, and vascular endothelial growth factor sets (Lee et al., 2022). Wilcoxon signed-rank tests were used to compare the groups.

### 2.10 Cell–cell communication analysis

The CellChat R package was used to visualize cell–cell interactions among immune cells, followed by the standard workflow and preprocessing steps. Then the potential interactions between ligands and receptors were explored based on the number of interactions and interaction strength.

### 2.11 Pseudotime trajectories and RNA velocity

Single-cell pseudotime trajectories were analyzed using the Monocle3 R package (ver. 1.2.0; https://cole-trapnell-lab.github.io/monocle3/). The “learn_graph” function was used to analyze the trajectories. Then the cells were organized in pseudotime using the “order_cells” function with a selected node that represents the hDPSCs to identify the pseudotime root node. RNA velocities were evaluated using the veloctyo.py v0.17.16 and scVelo algorithms (0.2.5.dev5+g1805ab4 [python 3.8.0]) (Bergen et al., 2020). The “Scv.tl.velocity” function was used to calculate the velocity. Velocity plots were visualized using the “scvelo.tl.velocity_embedding_stream (basis = ‘umap’)” function.

### 2.12 New RNA pathway analysis

The top 200 genes with the highest multiple difference of new and old RNA rates between the infection and control groups were selected to analyze pathway enrichment using the “Enrichr” tool (Kuleshov et al., 2016). Then we focused on the KEGG 2021 human pathway databases.

### 2.13 Statistical analysis

Statistical analyses were performed using Origin (2020b) and R (version 4.0.3) software. Statistical significance was assessed using an unpaired two-tailed Student’s *t* test, non-parametric Wilcoxon rank sum test, and analysis of variance. *p* values < 0.05 were considered indicative of statistical significance.

## 3 Results

### 3.1 Heterogeneity of hDPSCs infected with *P. gingivalis* and *E. faecalis*

**FIGURE 1:**
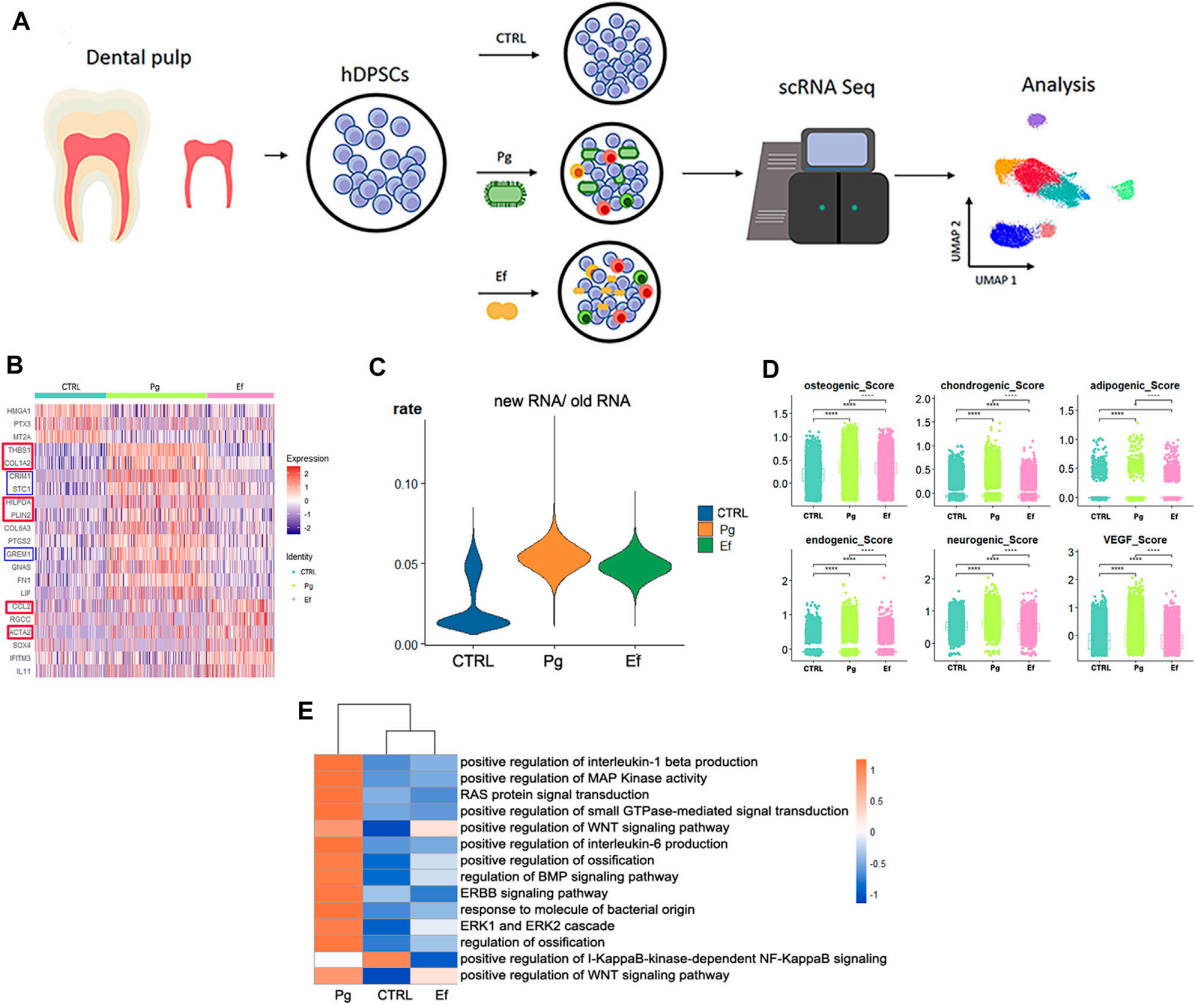
Study design and differential gene analysis. **(A)** Schematic graph showing the experimental design for single-cell RNA sequencing and analysis. **(B)** Heatmaps of the most differentially expressed genes among control, Pg, and Ef samples. Red and blue square frames indicated genes mentioned in the article. **(C)** Violin plots of the new RNA/old RNA rate of hDPSCs among each sample. **(D)** hDPSCs differentiation scores among three samples, including osteogenic, chondrogenic, adipogenic, endogenic, neurogenic, and VEGF scores. *p ≤ 0.05; **p ≤ 0.01; ***p ≤ 0.001; ****p ≤ 0.0001. **(E)** Heatmaps of enriched pathways among the three groups.

We used single-cell profiling to explore the cellular and molecular characteristics of hDPSCs infected with *P. gingivalis* or *E. faecalis* (Figure 1A). After removal of low-quality cells, 72,486 cells remained, including 21,736 non-infected cells (control sample), 30,599 cells infected with *P. gingivalis* (Pg sample), and 20,151 cells infected with *E. faecalis* (Ef sample). Supplementary Figure S1C is the bar graph of the cell populations.

We analyzed the differentially expressed genes (DEGs) among control, Pg, and Ef samples. The control cells expressed *HMGA1* and *MT2A*, which are involved in cell cycle regulation. The DEGs in the Pg sample were *THBS1*, *COL1A2*, *CRIM1*, and *STC1*, which are related to matrix formation and mineralization, and *HILPDA* and *PLIN2*, which are related to the cellular response to hypoxia. The Ef sample was characterized by *CCL2* expression, which is related to leukocyte chemotaxis, and *ACTA2*, which is related to actin (Figure 1B). The proportion of newly synthesized RNA in cells was increased after bacterial stimulation in the Pg and Ef samples (Figure 1C).

We conducted differentiation potential analysis of the three cell groups. The hDPSCs of the Pg sample showed the highest scores (*p* < 0.05), including the osteogenic, chondrogenic, adipogenic, endothelial, neurogenic, and VEGF scores (Figure 1D). These results suggest that hDPSCs could not maintain their stem cell status, but had multidirectional differentiation trends under conditions of *P. gingivalis* stimulation.

Figure 1E shows the differences in the expression of signaling pathways among the three cell groups. Certain signaling pathways were upregulated after *P. gingivalis* or *E. faecalis* infection, including the interleukin-1 beta, MAPK, WNT, ossification, BMP, extracellular signal-regulated kinase 1 (ERK1), and ERK2 pathways. The expression of the WNT signaling pathway is significantly upregulated by *P. gingivalis* or *E. faecalis* infection*.* The Pg sample showed significant upregulation of the BMP signaling pathway and ossification. The ERBB signaling pathway, RAS protein signal transduction, and small GTPase-mediated signal transduction were upregulated in Pg samples and downregulated in Ef samples. The I-KappaB-kinase-dependent NF-KappaB signaling pathway was significantly downregulated in the Pg and Ef samples (Figure 1E).

### 3.2 Classification of hDPSCs

**FIGURE 2:**
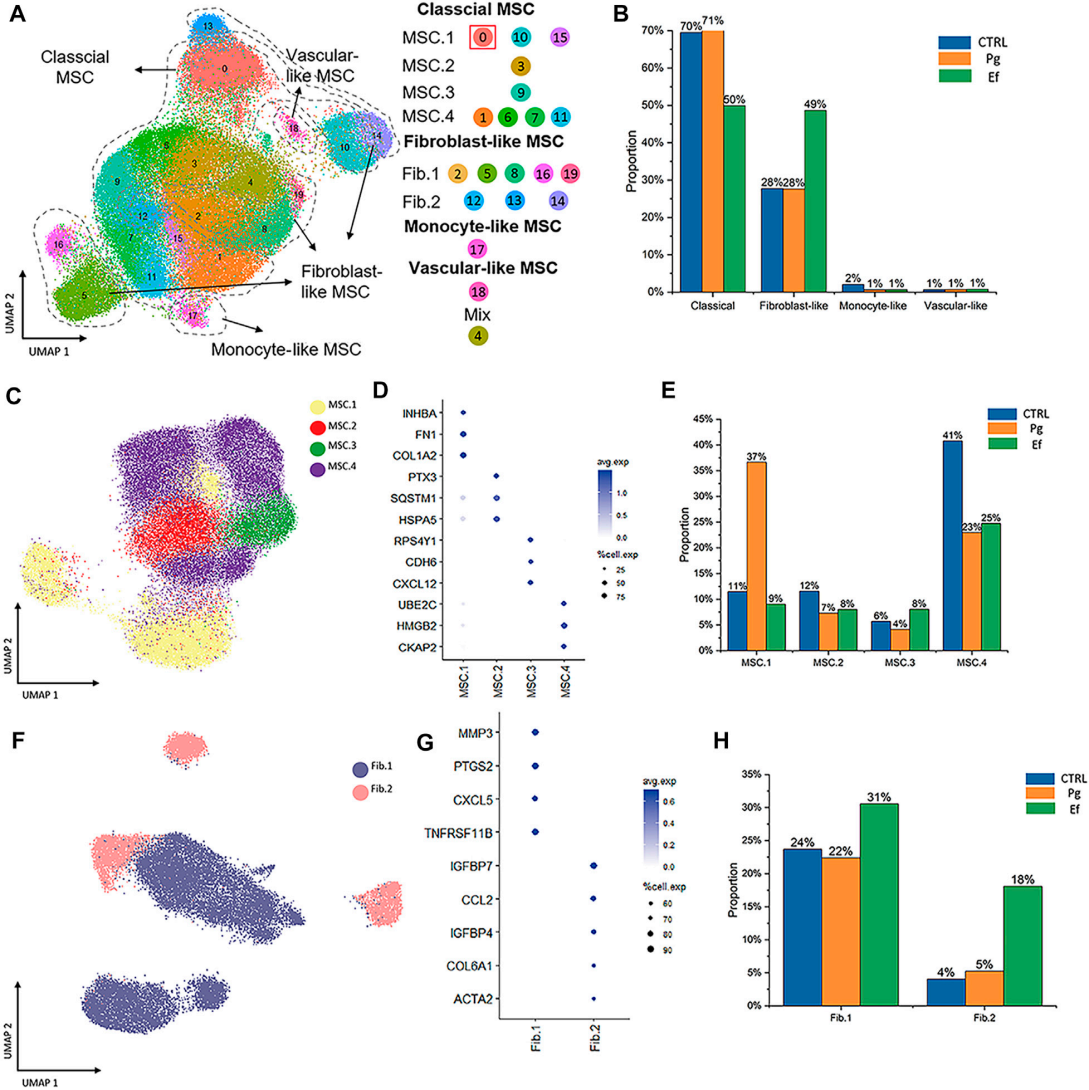
Cell types and clustering in the control, Ef, and Pg samples. **(A)** UMAP plot of the distribution of the 20 clusters. Clusters 0, 1, 3, 6, 7, 9, 10, 11, 15, and 20 were identified as MSC.1 (clusters 0, 10, and 15), MSC.2 (cluster 3), MSC.3 (cluster 9), and MSC.4 (clusters 1, 6, 7, and 11). Clusters 2, 5, 8, 12–14, 16, and 19 were fibroblast-like hDPSCs and included Fib.1 (clusters 2, 5, 8, 16, and 19) and Fib.2 (clusters 12–14). Cluster 17 included monocyte-like hDPSCs. Cluster 18 included perivascular-like hDPSCs. **(B)** Classical MSCs (46,902 cells), fibroblast-like hDPSCs (24,292 cells), monocyte-like hDPSCs (771 cells), and perivascular-like hDPSCs (160 cells) are shown. Proportion plots showing the percentage of four cell subclusters from each sample. **(C)** UMAP plot of classical MSC.1 (mineralization and ossification; 15,530 cells), MSC.2 (stress and inflammatory response; 6,367 cells), MSC.3 (osteoclastic reaction; 4,128 cells), and MSC.4 (cell cycle; 20,877 cells) populations. **(D)** Dot plots showing the expression of cluster-defining genes and percentage of cells expressing each gene in the four subtypes of classical MSC. The expression values are normalized and scaled averages. **(E)** Proportion plots showing the percentage of four subcluster types from MSCs. **(F)** UMAP plot of Fib.1 (inflammatory properties; 18,164 cells) and Fib.2 (myofibroblast-like hDPSCs; 6,128 cells) populations. **(G)** Dot plots showing the expression of cluster-defining genes and percentage of cells expressing each gene of the two subtypes of fibroblast-like hDPSCs. Expression values are normalized and scaled averages. **(H)** Proportion plots showing the percentage of two subcluster types from fibroblast-like hDPSCs.

We used unsupervised graph clustering to partition the cells into 20 clusters and visualized the clusters via uniform manifold approximation and projection (UMAP) (Figure 2A; Supplementary Figure S2). Subsequently, we used marker genes to identify four types of hDPSCs (Figure 2A), including classical mesenchymal stem cells (MSCs; *CD105*, *CD90*, and *CD73*), fibroblast-like hDPSCs (*DCN*, *COL1A1*, *COL3A1*, *LUM*, S100A4, *FAP*, *PDPN*, and *TPM1*), monocyte-like hDPSCs (*IFITM3*, *IFIT1*, *OASL*, and *MX1*), and perivascular-like hDPSCs (*ACTA2*, *MCAM*, *TAGLN*, and *PDGFRβ*) (Supplementary Figure S3). The four cell types were present in different proportions in each sample. The number of fibroblast-like hDPSCs was higher in the Ef sample than in control and Pg samples (49%, 28%, and 28%, respectively) (Figure 2B).

Next, we identified four subtypes of classical MSCs and two subtypes of fibroblast-like hDPSCs based on their marker genes and biological characteristics (Figure 2). Cluster 0, 10, and 15, which were identified as MSC.1, were linked to mineralization and ossification with expression of *INHBA*, *FN1*, and *COL1A2* (Figure 2D); their number was significantly higher in the Pg sample than in control and Ef samples (37%, 11%, and 9%, respectively) (Figure 2E). MSC.2 (cluster 3) was related to stress and the inflammatory response with expression of *PTX3*, *SQSTM1*, and *HSPA5* (Figure 2D). Because cluster 9 was associated with a unique gene ontology pathway, i.e., negative regulation of osteoblast differentiation and ossification, it was classified as MSC.3. MSC.4 included clusters 1, 6, 7, and 11 (Figure 2A).

Fib.1 included clusters 2, 5, 8, 16, and 19, and Fib.2 included clusters 12–14 (Figures 2A, F, G). The proportions of Fib.1 and Fib.2 were higher in Ef samples than control and Pg samples (Fib.1: 31%, 24%, and 22%, respectively; Fib.2: 18%, 4%, and 5%, respectively) (Figure 2H).

The above data showed that the distribution of hDPSCs under *P. gingivalis* or *E. faecalis* stimulation was obviously different (Figure 2).

Characteristics of and communication among classical MSC, fibroblast-like, monocyte-like, and perivascular-like hDPSCs

**FIGURE 3:**
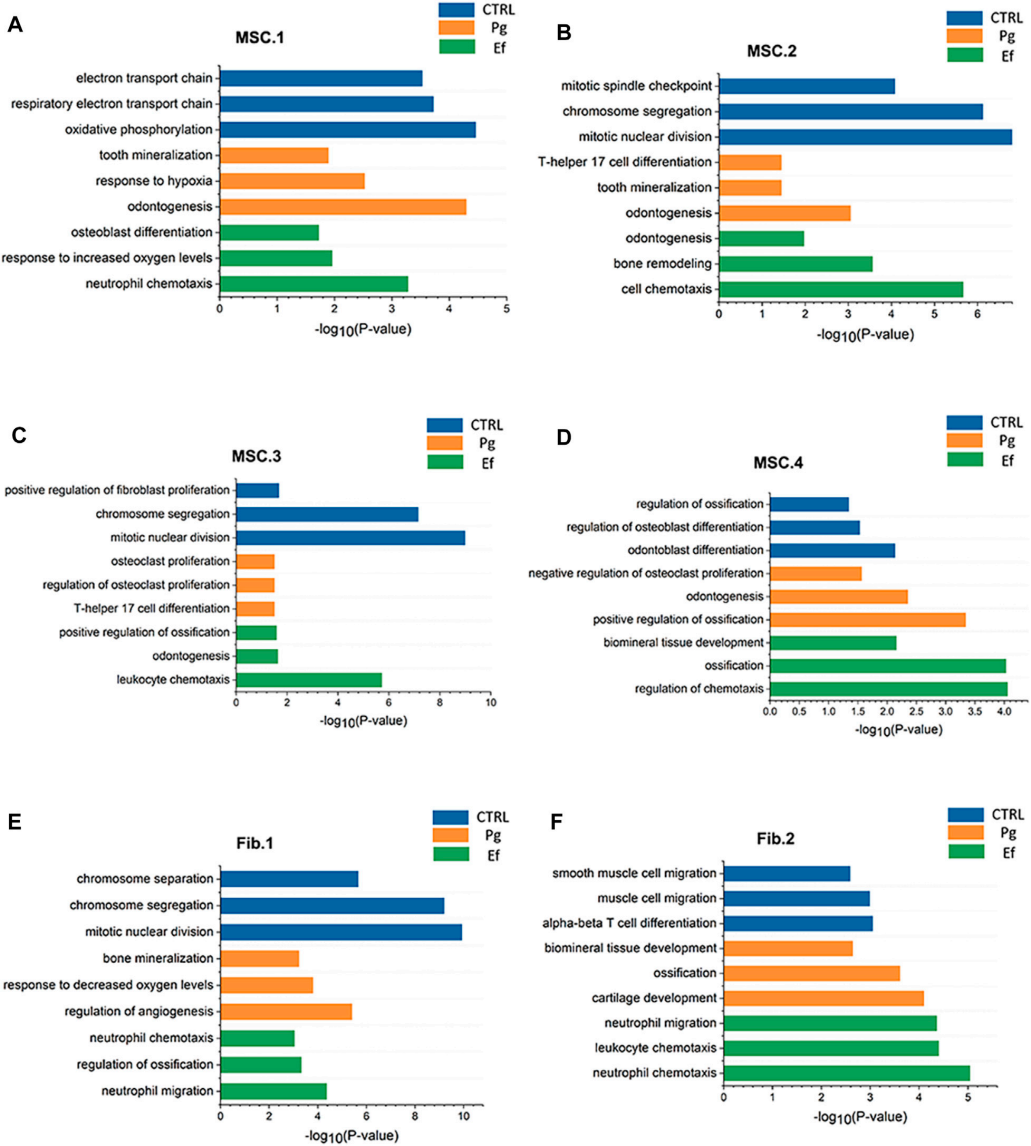
Pathway atlas for the characterization of classical MSCs and fibroblast-like hDPSCs. Bar charts of the most typical pathways enriched in MSC.1 **(A)**, MSC.2 **(B)**, MSC.3 **(C)**, MSC.4 **(D)**, Fib.1 **(E)**, and Fib.2 **(F)** subtypes from each sample. The length of the bar graph indicate the number of genes from each pathway.

The cell types had unique characteristics. Differential gene heatmaps of the cell clusters are shown in Supplementary Figure S4. MSC.1 was associated with mineralization and ossification and the expression of *INHBA*, *FN1*, and *COL1A2* (Figure 2D); there were also present at a significantly higher proportion in Pg samples than in other samples (Figure 2E), indicating significant odontogenic and osteogenic differentiation of hDPSCs after stimulation by *P. gingivalis*. The DEGs of MSC.1 were enriched in the cellular respiration and ion transport pathway in control samples. MSC.1 was associated with odontogenesis and response to the hypoxia pathway in Pg samples, and neutrophil chemotaxis and osteoblast differentiation pathways in Ef samples (Figure 3A).

MSC.2 (cluster 3) was related to the stress and inflammatory response associated with the expression of *PTX3*, *SQSTM1*, and *HSPA5* (Figure 2D). MSC.2 was associated with cell cycle function in control samples, odontogenesis, and the T-helper 17 cell differentiation pathway in Pg samples, and cell chemotaxis and bone remodeling in Ef samples (Figure 3B).

MSC.3 expressed genes related to osteoclasts (*RPS4Y1*, *CDH6*, and *CXCL12*) (Figure 2D). MSC.3 cluster genes were related to the regulation of fibroblast proliferation in control samples, osteoclast proliferation in Pg samples, and odontogenesis and positive regulation of ossification in Ef samples (Figure 3C).

MSC.4 expressed genes involved in the regulation of the cell cycle (*UBE2C*, *HMGB2*, and *CKAP2*) (Figure 2D). The MSC.4 genes were upregulated to a higher level in control samples than in Pg and Ef samples (Figure 2E). We speculated that, when hDPSCs are infected with *E. faecalis* and *P. gingivalis*, the cell cycle is affected and the stem cells differentiate into other cell types. MSC.4 was involved in the regulation of osteoblast differentiation and differentiation in control samples, positive regulation of ossification and negative regulation of osteoclast proliferation in Pg samples, and ossification and regulation of chemotaxis in Ef samples (Figure 3D). The increase in the odontogenic and osteogenic pathways in control samples indicated that hDPSCs can differentiate into odontoblasts and promote odontogenesis and osteogenesis without exogenous stimulation.

Fib.1 consisted of clusters 2, 5, 8, 16, and 19, and was associated with genes related to inflammatory and hypoxia-related gene expression (*MMP3*, *PTGS2*, *CXCL5*, and TNFRSF11B) (Figure 2G). The Fib.1 genes were associated with the cell cycle regulation in control samples, bone mineralization in Pg samples, and cell chemotaxis and ossification in Ef samples (Figure 3E).

Fib.2 are the characteristic effector cells stimulated by *E. faecalis* infection. The proportion of Fib.2 cells was significantly higher in Ef samples than in the other two samples, which had few Fib.2 cells (Figure 2H). Similar to myofibroblasts, these cells showed upregulated *ACTA2* expression (Figure 2G) and enrichment of the muscle cell migration signaling pathways (Figure 3F).

Monocyte-like hDPSCs exhibited upregulation of genes involved in immune responses. Perivascular-like hDPSCs showed similar characteristics to perivascular cells, and a similar pattern of gene expression across the control, Pg, and Ef samples as for monocyte-like hDPSCs (Supplementary Figure S4). The samples had similar proportions of monocyte-like and perivascular-like hDPSCs.

### 3.3 Differentiation potency scores of hDPSCs subtypes

**FIGURE 4:**
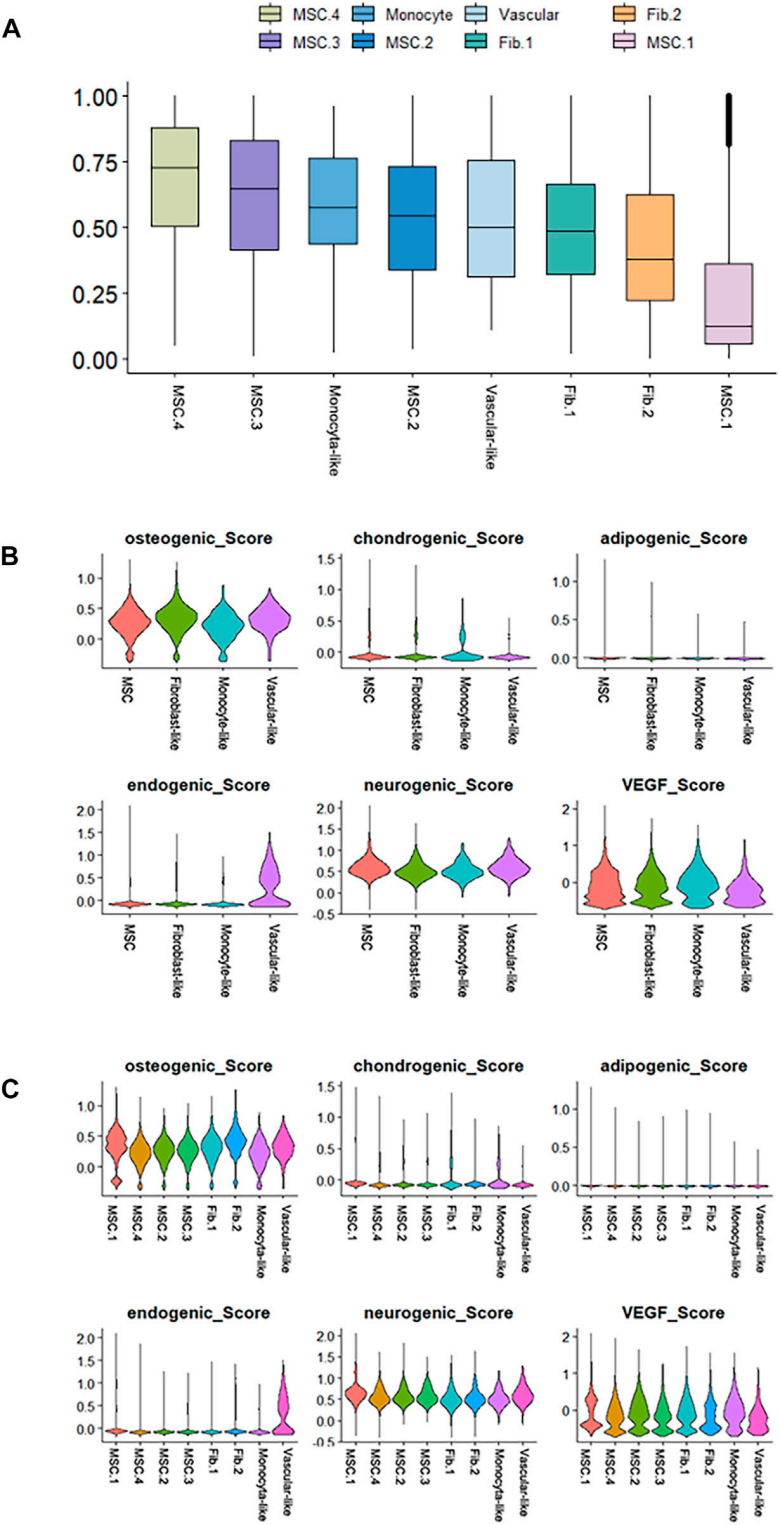
Differentiation potency scores. **(A)** Box diagrams showing the overall differentiation potential of various cell types. **(B)** Violin plots showing the hDPSCs differentiation scores among the four cell types. **(C)** Violin plots showing the hDPSCs differentiation scores among the subtypes.

Next, we performed cell differentiation potential analyses of the cell clusters. MSC.4 had the highest differentiation potential, whereas MSC.1 had the lowest, indicating that MSC.1 was relatively active in the late stages of differentiation (Figure 4A). Perivascular-like hDPSCs had a higher endogenic score (Figure 4B). The osteogenic score of MSC.1 was higher than that of other MSC clusters (Figure 4C).

### 3.4 Pseudotime analysis of hDPSCs-generated differentiation trajectories

We used the Monocle3 R package and scVelo to reconstruct the fate decisions and pseudotime trajectories of hDPSCs and explore the gene-specific transcriptional dynamics of hDPSCs over time.

**FIGURE 5:**
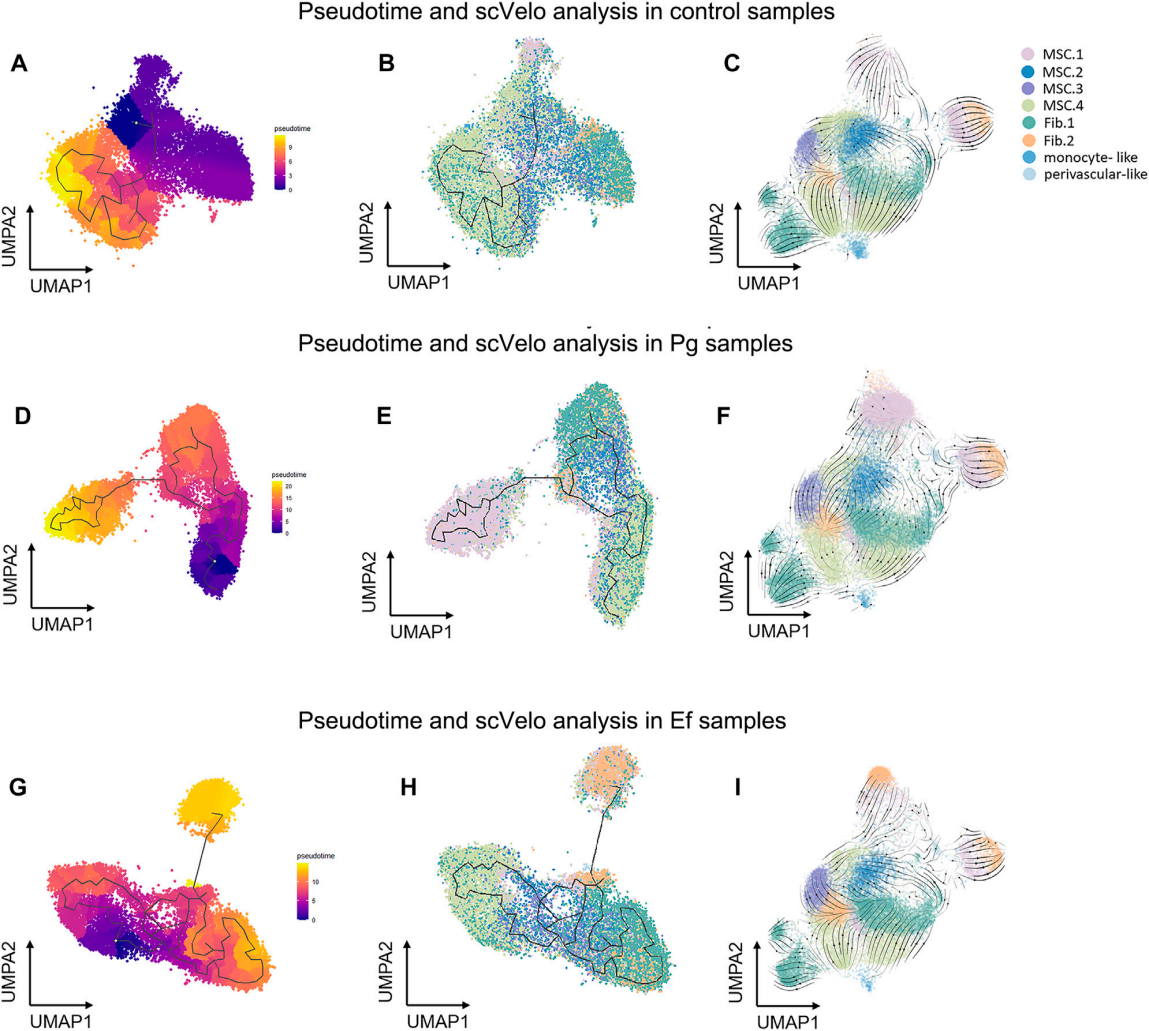
Pseudotime and scVelo analysis of hDPSCs subtypes. **(A** , **D**, **G)** UMAP plots showing branched cell trajectories (black lines) and cell type in control **(A)**, Pg **(D)**, and Ef **(G)** samples. **(B**, **E**, **H)** UMAP plots showing hDPSCs colored by pseudotime in control **(B)**, Pg **(E)**, and Ef **(H)** samples. **(C**, **F** , **I)** UMAP plots showing the velocity trajectory across hDPSCs subtypes in control **(C)**, Pg **(F)**, and Ef **(I)** samples. The arrows indicate the predicted future cell states.

Three hDPSCs samples were analyzed (Figure 5). We selected classical MSC.4 (cell cycle) as the root principal point because of its role in the cell cycle. The analysis of cell differentiation potential showed that MSC.4 was relatively active in the early stages of differentiation. The UMAP plot exhibited different patterns for each sample. In the control samples, fibroblast-like hDPSCs were ordered by later pseudotime. In Pg samples, hDPSCs related to the cell cycle evolved into classical MSC.1 (mineralization and ossification), whereas in Ef samples, MSC.4 developed into fibroblast-like hDPSCs (Figure 5).

### 3.5 Cellular communications in hDPSCs

**FIGURE 6:**
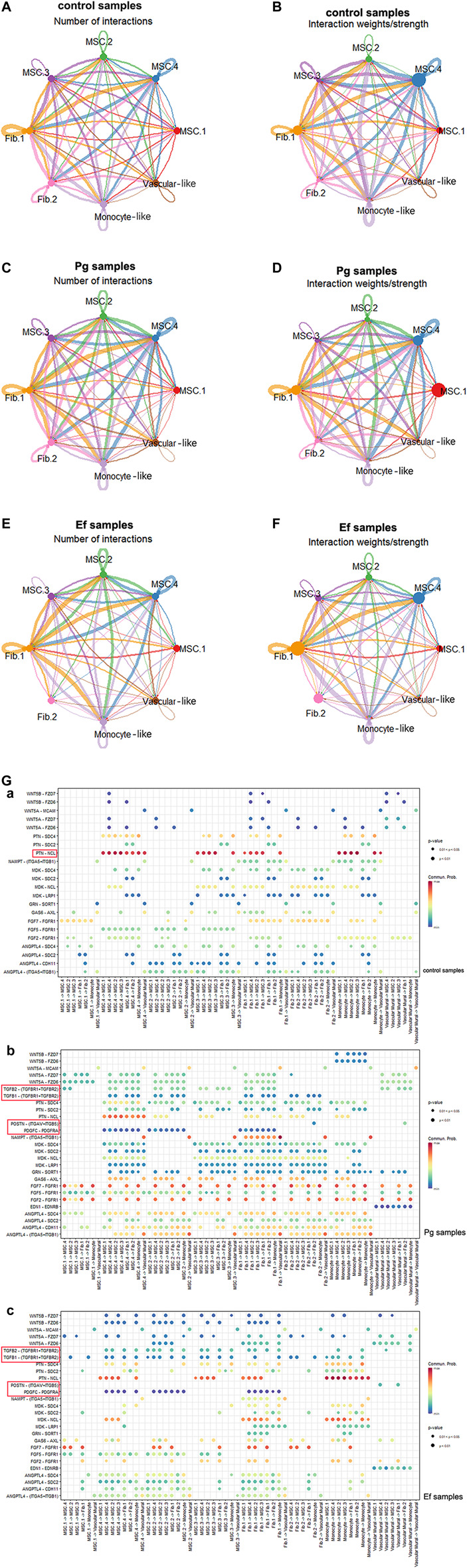
Cell–cell interactions among different subtypes. **(A** , **C** , **E)** Circle plots showing the number of cell–cell interactions in control **(A)**, Pg **(C)**, and Ef **(E)** samples. The edge width is proportional to the indicated number of ligand–receptor pairs. **(B** , **D** , **F)** Circle plots showing the weight/strength of cell–cell interactions in control **(B)**, Pg **(D)**, and Ef **(F)** samples. The edge width is proportional to the indicated weight/strength of ligand–receptor pairs. **(G)** Dot plots showing the ligand–receptor expression among various cell types. **(a)**, **(b)**, **(c)**.

The cellular communications of hDPSCs are involved in multiple metabolic processes and biological functions. Using the CellChat R package, we analyzed the mutual relationships of eight cell subtypes via ligand–receptor interactions, which regulate hDPSCs differentiation and development. Bacterial stimulation promotes communication among cells, particularly between MSC.2 (stress and inflammatory response) and monocyte-like hDPSCs, indicating activation of the immune inflammatory response. Fib.2 myofibroblasts and perivascular-like hDPSCs were relatively independent of each other (Figure 6; Supplementary Figure S6), indicating that they represented late stages of differentiation.

Analyses of ligand–receptor interactions that regulate hDPSC differentiation and development showed similar results. In control samples, there were significant cellular interactions among cell groups, as well as the presence of ligand receptors among different groups (Figure 6G). PTN-NCL was highly expressed in control samples but significantly downregulated in Pg samples (Figure 6H). The TGF-related ligand receptors, POSTN-(ITGAV + ITGB5) and PDGFC-PDGFRA were activated in response to *P. gingivalis* and *E. faecalis* infection*.* These ligand receptor pairs may be related to cell migration and adhesion. Additionally, we observed enhanced intercellular interactions between FGF and ANGPTL, which are related to angiogenesis.

BMP5-(ACVR1+ACVR2A), BMP7-(ACVR1+ACVR2A), and BTLA-TNFRSF14 were only detected in control samples. In the Pg sample, we observed interactions between ADM and CALCRL, which is related to periodontitis; EDA and EDA2R, which is related to ectodermal development; and SPP1 and CD44 and SPP1 and (ITGA4+ITGB1), which promotes phosphoprotein secretion and regulates bone salinization. The Ef samples also showed interactions between C3 and C3AR1 and HC and C5AR1, which are associated with complement activation; and SST and SSTR2, which negatively regulates cell proliferation (Supplementary Table S1).

### 3.6 hDPSCs mineralization attenuates *P. gingivalis* stimulation

**FIGURE 7:**
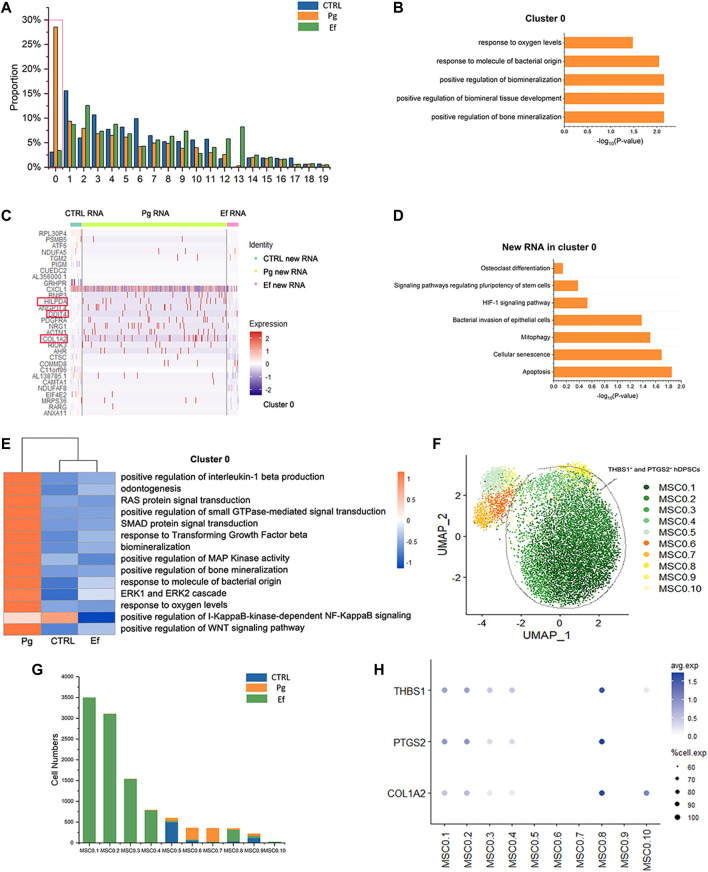
Responses of hDPSCs to *P. gingivalis* infection. **(A)** Proportion plots showing the percentage of 20 subclusters from each sample. Red line used to mark the cluster 0, the vast majority of cells in the 0 clusters originate from Sample Pg. **(B)** Bar chart of pathways enriched in cluster 0. The length of the bar graph indicates the number of genes from each pathway. **(C)** Heatmap of the most differentially expressed genes of new RNA in cluster 0 from each sample. **(D)** Bar chart of new RNA pathways enriched in Cluster 0. The length of the bar graph indicates the number of new genes from each pathway. **(E)** Heatmap of pathways enriched in cluster 0 among the three groups. **(F)** UMAP plot depicting cluster 0.1–0.10 populations in cluster 0. **(G)** Number of cluster 0.1–0.10 cells. **(H)** Dot plots showing the expression of *THBS1*, *PTGS2*, and *COL1A2* among clusters 0.1–0.10.

We analyzed 20 small clusters of cells, and found different trends among different samples for each cluster (Figure 7). Interestingly, the cell percentage of cluster 0 was nearly 10-fold higher in Pg samples than in control and Ef samples. The control and Ef samples had a similar number of cells in cluster 0 (Figure 7A), indicating that cluster 0 cells are the effector cells against *P. gingivalis* stimulation. DEGs in Cluster 0 were related to the positive regulation of biomineralization and response to the hypoxia pathway in the Pg samples (Figure 7B). This cell cluster exhibited increased levels of new RNA and upregulated expression of *HILPDA* and *DDIT4*, which are closely related to the cellular response to hypoxia; and *COL1A2*, which is closely related to cell mineralization (Figures 7C, D).

The pathways enriched in cluster 0 of hDPSCs included the hypoxia response, mineralization, stem cell maintenance, and osteoclasts (Figures 7B, D). Changes were detected in several signal transduction factors, including positive regulation of pathway-restricted SMAD protein phosphorylation, positive regulation of Ras protein signal transduction, ERK1 and ERK2 cascades, and the TGF-beta signaling pathway, as well as negative regulation of the I-KappaB-kinase-dependent NF-KappaB signaling pathway (Figure 7E). These findings suggest the involvement of the classical signaling transduction pathway and represent the response of hDPSCs to *P. gingivalis* invasion.

We further analyzed cluster 0 cells, divided them into 10 new clusters (Figure 7F), and screened cells resistant to *P. gingivalis* infection. MSC0.1, MSC0.2, MSC0.3, MSC0.4, and MSC0.8 were present in Pg samples (Figure 7G) but not in control or Ef samples. These results suggest that the cells produced by *P. gingivalis* stimulation are characterized by high expression levels of *THBS1*, *PTGS2*, and *COL1A2* (Figure 7H). These cells were categorized as *THBS1* ^*+*^ and *PTGS2* ^*+*^ hDPSCs.

### 3.7 hDPSCs differentiate into fibroblast-like cells after *E. faecalis* stimulation

The Ef samples were characterized by expression of *CCL2*, which is related to leukocyte chemotaxis, and *ACTA2*, which is an actin-related gene (Figure 1B).

Fib.1 and Fib.2 had higher proportions in Ef samples than in Pg and control samples (Fib.1: 31%, 22%, and 24%, respectively; Fib.2: 18%, 5%, and 4%, respectively) (Figure 2H). These results indicate that hDPSCs differentiate into fibroblast-like cells after *E. faecalis* infection. Fibroblasts and myofibroblasts play a key role in the defense of dental pulp against invasion by *E. faecalis*. Pseudotime analysis also showed that hDPSCs differentiated into fibroblasts and myofibroblasts.

## 4 Discussion

**FIGURE 8:**
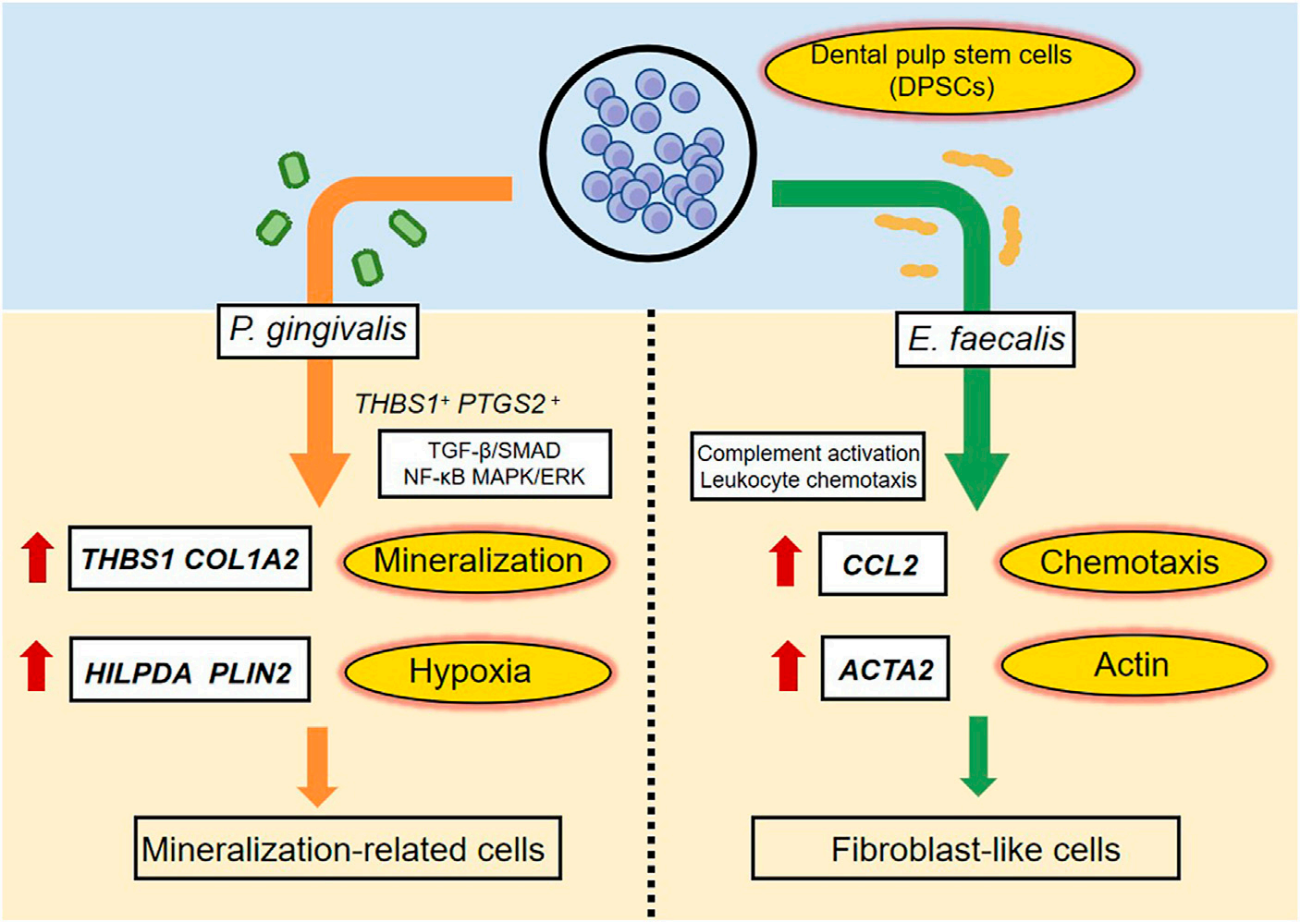
A schematic diagram recapitulating the response of hDPSCs to *P. gingivalis* or *E. faecalis.* When hDPSCs infected by *P. gingivalis, THBS1* and *COL1A2,* which are related to matrix formation and mineralization, and *HILPDA* and *PLIN2*, which are related to the cellular response to hypoxia were the most differentially expressed genes. *THBS1* ^*+*^ *PTGS2* ^*+*^ hDPSCs prevented *P. gingivalis* infection by regulating the TGF-β/SMAD, NF-κB, and MAPK/ERK signaling pathways. hDPSCs infected by *P. gingivalis* undergo multidirectional differentiation, particularly to the mineralization-related cell lineage. The Ef samples were characterized by the expression of *CCL2*, which is related to leukocyte chemotaxis, and *ACTA2*, which is related to actin. hDPSCs mediated complement activation and leukocyte chemotaxis pathways. The presence of *E. faecalis* promoted the differentiation of hDPSCs into fibroblast-like cells.

We evaluated the effects of *P. gingivalis* and *E. faecalis* on hDPSCs in a bacteria–cell coculture model using single-cell sequencing. The hDPSCs had different responses to the two types of bacteria in terms of the cell clusters mobilized and their differentiation. After *P. gingivalis* stimulation, hDPSCs showed matrix mineralization-related characteristics, whereas *E. faecalis* caused the cells to differentiate into fibroblast-like cells. These findings provide insight into the mechanisms underlying the responses of hDPSCs to invasion by distinct bacterial species and the effects of residual bacteria on RET outcomes. Figure 8 summarized the highlights of the findings.

In response to *P. gingivalis* stimulation, a cluster of cells characterized by high expression levels of thrombospondin 1 (*THBS1*) and prostaglandin-endoperoxide synthase 2 (*PTGS2*) (i.e., *THBS1* ^*+*^ and *PTGS2* ^*+*^ hDPSCs) were identified. In a previous study, the level of TSP1 protein encoded by *THBS1* was increased in gingival tissues of patients with periodontitis and were upregulated by Pg-LPS (Gokyu et al., 2014). The TSP1 protein is highly expressed in and secreted by odontoblasts; it is involved in biomineralization and odontogenesis (Ueno et al., 1998). *PTGS2*, also known as cyclooxygenase, is responsible for the prostanoid biosynthesis involved in inflammation and mitogenesis. *P. gingivalis* is associated with increased *PTGS2* expression, *PGE2* secretion (Symmank et al., 2021), and the development of pulpitis. In the present study, the high expression levels of *THBS1* and *PTGS2* in the Pg samples suggest that hDPSCs stimulate the immune inflammatory response against bacteria and prevent *P. gingivalis* invasion through mineralization and repair.

In cluster 0, the TGF-β and WNT signaling pathways were upregulated (Figure 7E). These conservative signaling pathways regulate multiple cellular functions, including cell growth, adhesion, migration, cell-fate determination, differentiation, and apoptosis (Kubiczkova et al., 2012). These pathways play a key role in the synthesis of dentin proteins and proteases (Niwa et al., 2018). Additionally, positive regulation of pathway-restricted SMAD protein phosphorylation was found to be enriched. Hwang et al. (2008) reported that TGF-β is released during reparative dentin formation and activates the Smad proteins to control TGF-β cell signaling. In Pg samples, the enrichment of TGF β and SMAD may be related to the formation of repair tissue. The regulation of the ERK1 and ERK2 cascades and small RAS GTPase were enriched in Cluster 0. The ERK1 and ERK2 proteins belong to a family of structurally related kinases called mitogen-activated protein kinases (MAPKs), and are essential components of the TGF-β signaling pathway and mitogenic signals largely channeled by small RAS GTPases. This pathway is an important therapeutic target for inflammatory diseases and can induce osteogenic gene expression (Lavoie et al., 2020). The MAPK/ERK pathways are activated to regulate inflammation through downstream RAS GTPase.

The signaling pathway of inhibitor of nuclear factor kappa-B (IκB) was suppressed (Figure 7E), suggesting upregulation of nuclear factor kappa-B (NF-κB) expression. NF-κB is associated with multiple biological reactions, cytokines, chemokines, cell adhesion molecules, growth factors, and immune receptors (such as IL-1β and PTGS2, which were detected in our study in Figures 7E, H) (Paudel et al., 2014). NF-κB and MAPK pathways are activated by the LPS in dental pulp cells (Kim et al., 2012; Lee et al., 2022). These results suggest that hDPSCs resist *P. gingivalis* invasion by regulating the TGF-β/SMAD, NF-κB, and MAPK/ERK pathways.

The differentiation potency scores, including osteogenic, chondrogenic, adipogenic, endothelial, neurogenic, and VEGF factors, showed the multi-directional differentiation potential of hDPSCs of the Pg samples (Figure 1D). However, after *P. gingivalis* stimulation, the cells differentiated into terminal cells (MSC.1) related to mineralization expression. In Pg samples, the number of MSC.1 cells was increased (Figure 2E), and pseudotime analysis showed differentiation of hDPSCs into MSC.1 cells (Figure 5). The genes that were significantly overexpressed in Pg samples (*STC1*, *CRIM1*, and *GREM1*) (Figure 1B, blue square frame marked) were involved in the regulation of matrix mineralization and bone formation (Yoshiko et al., 2002; Liu et al., 2021).

Interestingly, the presence of bacteria was associated with altered expression of hypoxia-related genes, although the coculture was not performed in a hypoxic environment. The highly expressed genes *HILPDA*, *DDIT4*, and *HIF1A* are closely related to the cellular response to hypoxia (Figure 1B). Previous studies have shown that Pg-LPS and hypoxia promote periodontal tissue destruction (Gölz et al., 2015). Hypoxia also enhances the odontogenic and osteogenic properties of hDPSCs through the HIF1α-Wnt/β-catenin signaling pathway (Orikasa et al., 2022). Based on our classification, the enrichment of hypoxic and hyperoxic pathways in Fib.1 and MSC.1 (Figure 3) indicates that these cell types respond to changes in the oxygen level.

Multiple cell chemotaxis-related and complement-activated signaling pathways were detected in the Ef samples, whereas the corresponding pathways were not significantly enriched in the Pg samples (Figure 3). These findings indicate an immune escape effect of *P. gingivalis*. *P. gingivalis* is internalized into cells through MAPK activation to avoid extracellular clearance, inhibit IL-8 production by serine dephosphorylation of the p65 subunit of NF-κB, and reduce neutrophil migration and killing. *P. gingivalis* cleaves the complement component C3 into C3a and C3b, and degrades them to disrupt the complement cascade reaction and inhibit T cell activation to avoid being killed by the host’s immune response (Zheng et al., 2021). However, previous studies have mainly focused on periodontal tissue. The present study found that immune evasion can also occur during dental pulp inflammation and pulp regeneration.

The numbers of fibroblast-like cells were significantly increased after *E. faecalis* infection, including typical myofibroblasts (Fib.2) with high *ACTA2* expression (Figure 2). In contrast to the changes in Ef samples, hDPSCs after *P. gingivalis* infection did not differentiate into fibroblast-like cells (Figure 2B). These differences may be due to the different characteristics of the bacteria. The hDPSCs that were exposed to *E. faecalis* differentiated into fibroblast-like cells via chemotaxis of leukocytes and phagocytes, and activation of the complement system. Dental pulp fibroblasts play pivotal roles in the production of the complement system proteins involved in defense processes, control of inflammation, dentin-pulp regeneration, and dental pulp wound healing (Tsai et al., 2022). The high activity of the leukocyte migration and complement pathways in the Ef samples in our study are consistent with these findings (Figure 3). Myofibroblasts in the dental pulp originate from MSCs (Dimitrova-Nakov et al., 2014) and promote extracellular matrix remodeling in damaged dental pulp (Edanami et al., 2017) and tissue repair through collagen synthesis. Some myofibroblasts produce newly differentiated odontoblast-like cells that synthesize restorative dentin (Álvarez-Vásquez & Castañeda-Alvarado, 2022). Our study showed that myofibroblasts play a significant role in tissue repair after *E. faecalis* invasion.

The different responses to the two types of bacteria may be related to their different characteristics, in terms of aspects such as oxygen demand and virulence. LPS and lipoteichoicacid (LTA) have different molecular structures and antigens, as well as recognition patterns, leading to different responses against bacteria (Colombini-Ishikiriama et al., 2020). The upregulation of the TLR/MyD88/NF-κB pathway of LPS-treated hDPSCs is associated with increased interleukin production and odontoblastic differentiation (Brodzikowska et al., 2022). LTA-stimulated hDPSCs release IL-6 and IL-8 in a dose-dependent manner and promote cell proliferation, cell migration, and the local inflammatory response through cytokine release; however, LTA does not influence osteogenic or odontoblastic differentiation (Shayegan et al., 2021). In our study, the effects of the two virulence factors on the cells were consistent with those of the two bacteria on hDPSCs.

Based on previous studies, the tissues that formed in the canals of revascularized/revitalized teeth were identified as fibrous connective tissue, similar to that found in the periodontal ligament and cementum-like or bone-like tissue. Fibrous connective tissue, which is mainly characterized by fibroblasts and collagen fibers, filled the radiographically empty canal space; no odontoblast-like cells were observed, particularly in the area of severe inflammation (Becerra et al., 2014; Nosrat et al., 2015). The hDPSCs could differentiate into odontoblast-like cells and express dentin sialophosphoprotein (*DSPP*) and osteocalcin (*OCN*), which are used to regenerate dentin (Alongi et al., 2010; Liao et al., 2011). However, the corresponding genes were not detected in our study, which may be related to the presence of bacteria. Some studies have shown that *P. gingivalis* suppresses the expression of *DSPP* and *OCN* in hDPSCs (Yamagishi et al., 2011), suggesting a negative effect on pulp regeneration. We did not detect cementogen-related genes (*CDGF*, *CAP*, and *CEMP1*), which may be due to the limited stimulation time, as we only observed early reactions.

In conclusion, hDPSCs do not maintain their stem cell status in the presence of *P. gingivalis* and *E. faecalis* (Figure 4A). The proportion of MSC.4 with the highest differentiation potential score (Figure 4A) decreased after bacterial invasion and differentiated into MSC.1or Fib.2 (Figure 5), whose differentiation potential are lower than MSC.4. They differentiate into mineralization-related cells in the presence of *P. gingivalis* and into fibroblast-like cells in the presence of *E. faecalis*. Additionally, we identified certain hDPSC clusters and signaling pathways that respond to each type of bacteria. These findings enhance our understanding of the molecular mechanisms underlying the responses of dental pulp to bacterial invasion. Future studies should create a microenvironment that is conducive to the survival and directional differentiation of stem cells during RET.

### 4.1 Limitations

Although scRNA-seq is useful for determining the mechanisms underlying the responses of hDPSCs to various bacterial infections, our findings need to be validated in future studies.

## Data Availability

The original contributions presented in the study are included in the article/Supplementary Material, further inquiries can be directed to the corresponding author FC (chenfeng2011@hsc.pku.edu.cn).

## Supplementary material

SUPPLEMENTARY FIGURE S1 Quality control of samples **(A)** Violin plots showing the number of features, RNA counts, and percent mitochondrial transcripts in the control, Pg, and Ef samples prior to quality control. **(B)** Violin plots showing the number of features, RNA counts, and percent mitochondrial transcripts following quality control. **(C)** Bar graphs showing the number of cells before (preQC) and after (postQC) quality control in each sample.

SUPPLEMENTARY FIGURE S2 Differentially expressed genes for the 20 cell clusters. Heatmap plot showing the average expression of the top differentially regulated genes for the 20 cell clusters.

SUPPLEMENTARY FIGURE S3 Identification of cell types. Feature plots (UMAP) displaying the main signatures for each subtype of classical MSC **(A)**, fibroblast-like hDPSCs **(B)**, perivascular-like hDPSCs **(C)**, and monocyte-like hDPSCs **(D)**.

SUPPLEMENTARY FIGURE S4 Genes expressed in MSC.1, MSC.2, MSC.3, MSC.4, Fib.1, Fib.2. Heatmap of the expression of the most differentially expressed genes among the three samples in MSC.1**(A)** , MSC.2 **(B)**, MSC.3 **(C)**, MSC.4 **(D)**, Fib.1 **(E)** and Fib.2 **(F)**.

SUPPLEMENTARY FIGURE S5 Genes expressed in monocyte-like and perivascular-like hDPSCs. Heatmaps showing the expression of the most differentially expressed genes between each cluster compared to the other clusters in monocyte-like hDPSCs **(A)** and perivascular-like hDPSCs **(B)**.

SUPPLEMENTARY FIGURE S6 Cell–cell interactions between different subtypes. **(A, C, E)** Circle plots showing the number of cell–cell interactions in control **(A)**, Pg **(C)**, and Ef **(E)** samples. The edge width is proportional to the indicated number of ligand–receptor pairs. **(B, D, F)** Circle plots showing the weight/strength of cell–cell interactions in control **(B)**, Pg **(D)**, and Ef **(F)** samples. The edge width is proportional to the indicated weight/strength of ligand–receptor pairs.

SUPPLEMENTARY TABLE S1 Ligand-receptors pairs in control, Pg, and Ef samples.
