# Supplementary figures and images for: Single-cell atlas of dental pulp stem cells exposed to the oral bacteria Porphyromonas gingivalis and Enterococcus faecalis
Source: Front Cell Dev Biol. 2023 May 23;11:1166934. doi: 10.3389/fcell.2023.1166934 (PMC10242116; doi:10.3389/fcell.2023.1166934)

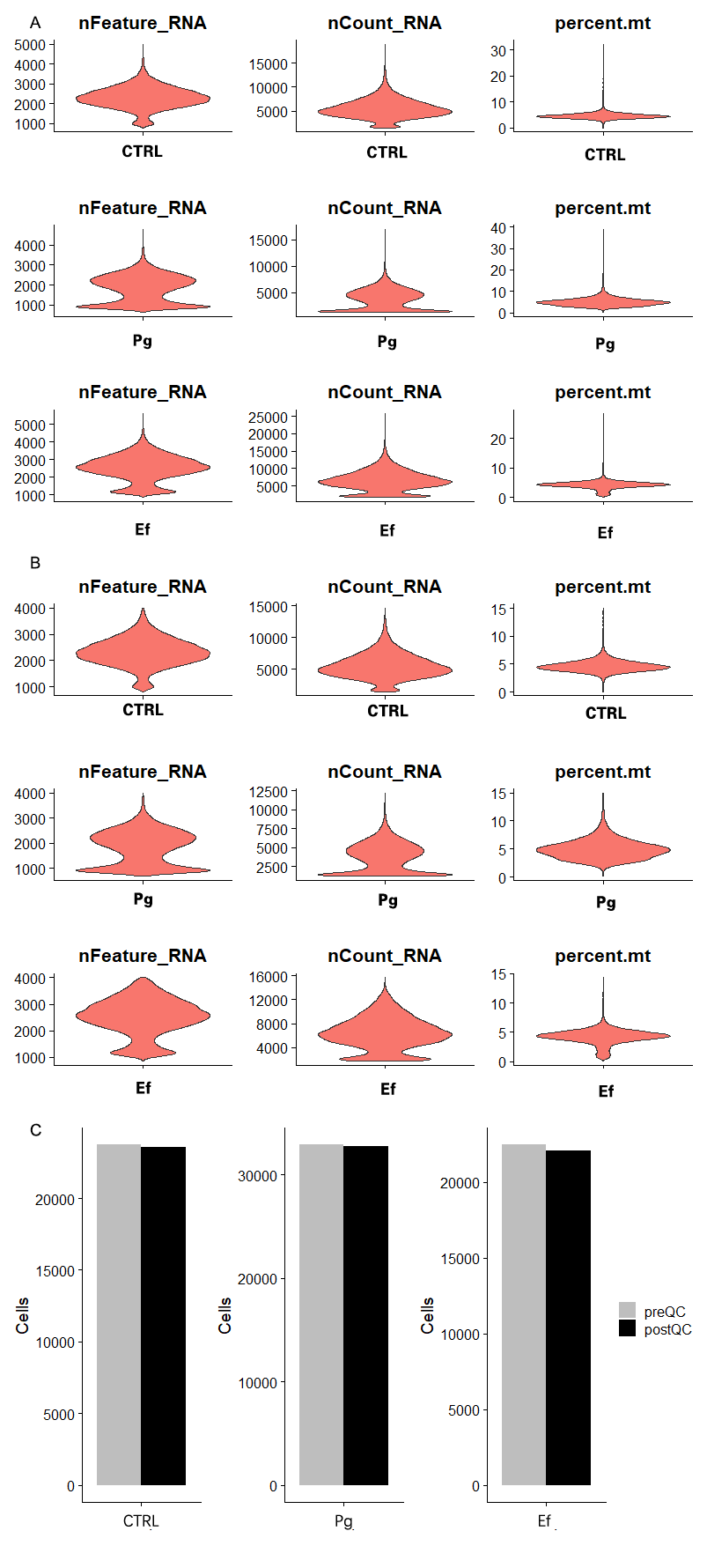

Supplement: Supplementary file 2 [file DataSheet3.ZIP › supplement figure/FigS1(A).tiff]

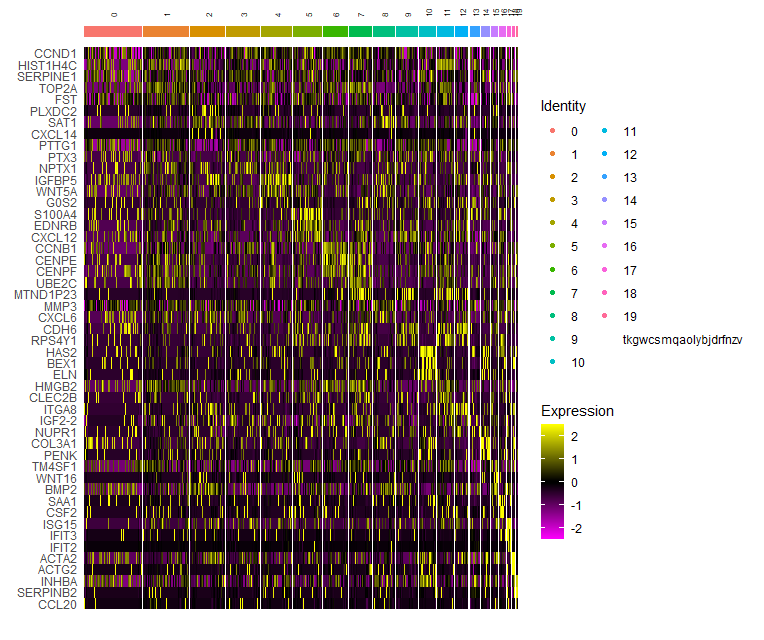

Supplement: Supplementary file 2 [file DataSheet3.ZIP › supplement figure/FigS2.tiff]

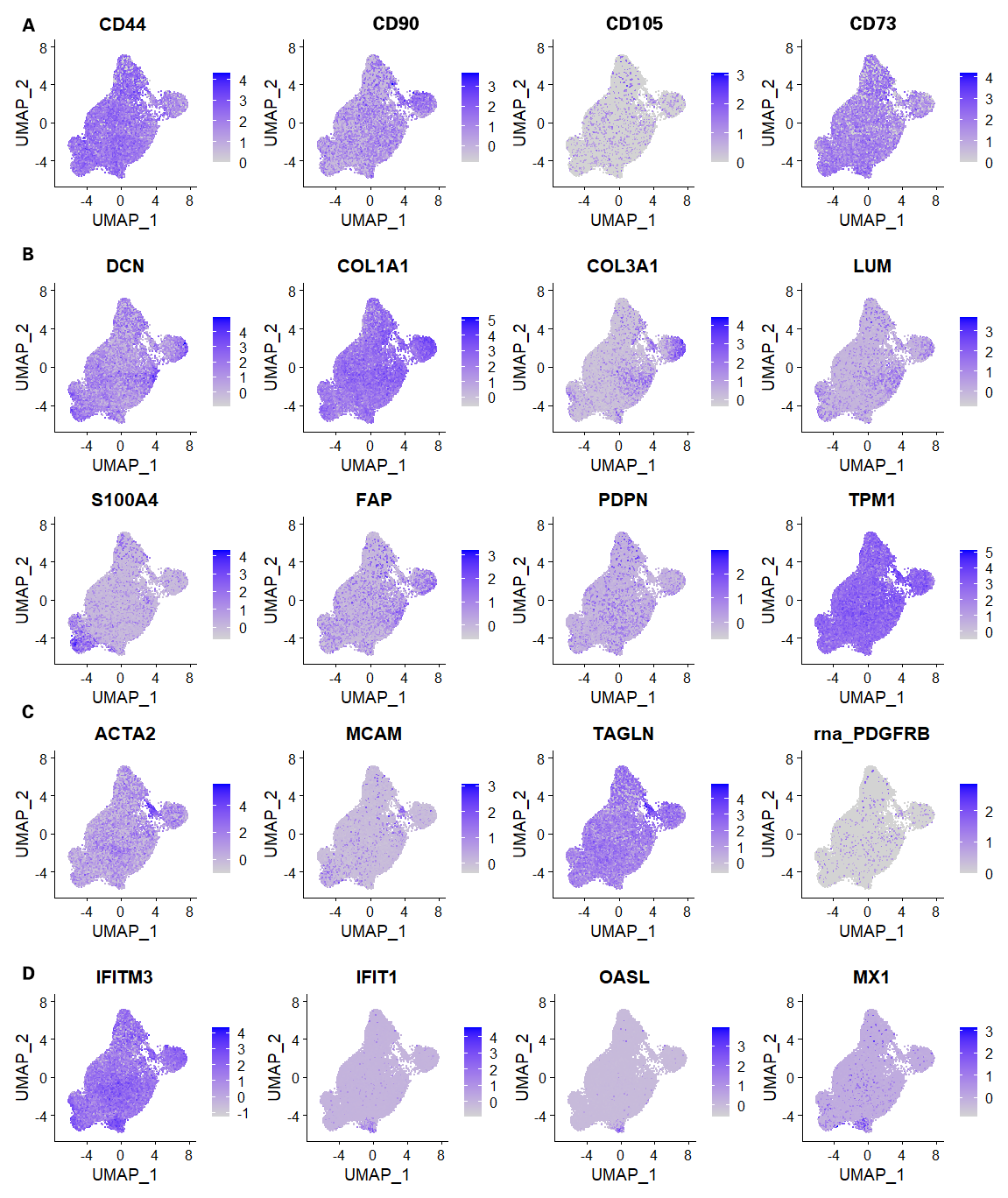

Supplement: Supplementary file 2 [file DataSheet3.ZIP › supplement figure/FigS3(A).tiff]

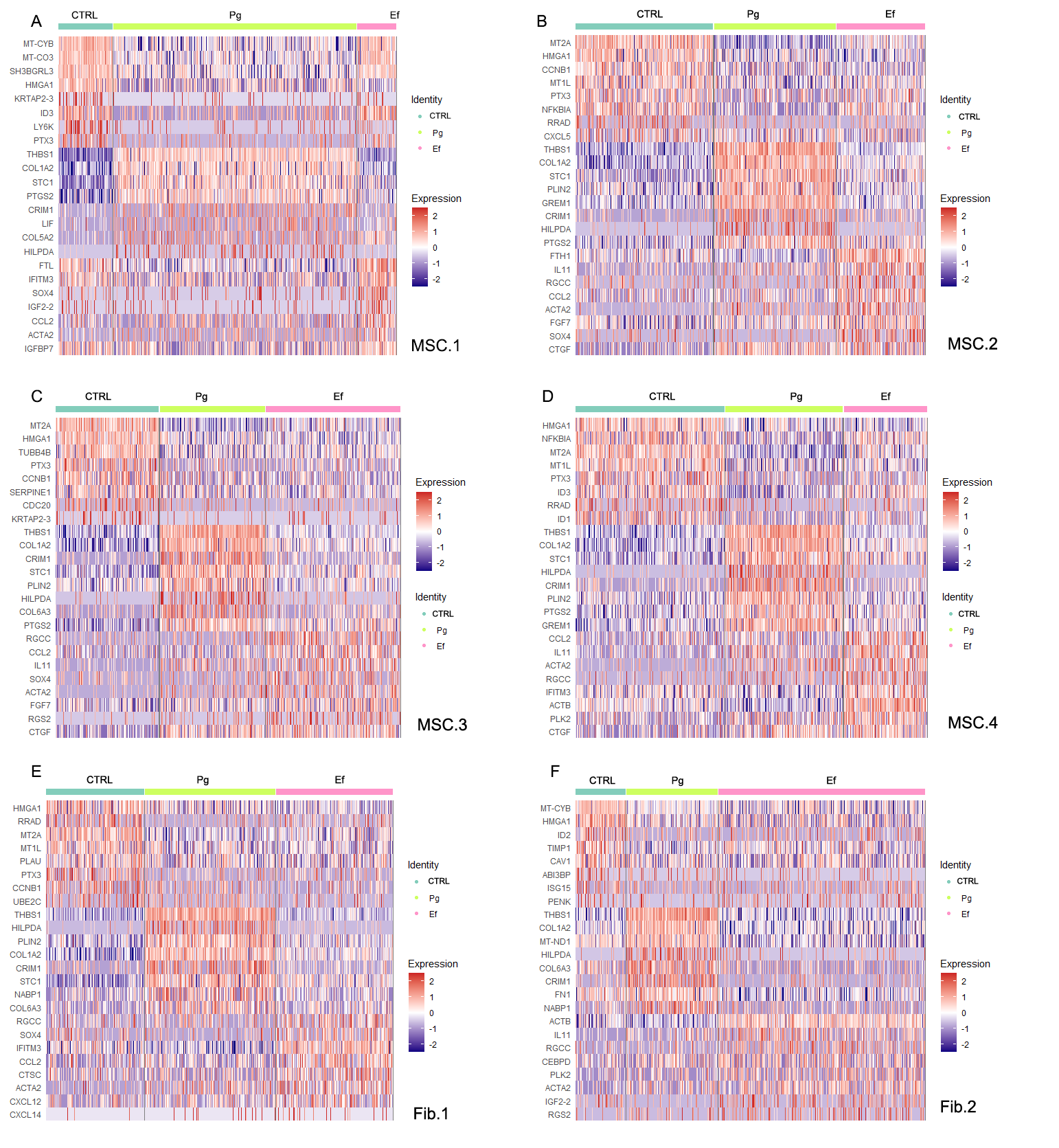

Supplement: Supplementary file 2 [file DataSheet3.ZIP › supplement figure/FigS4(A).tiff]

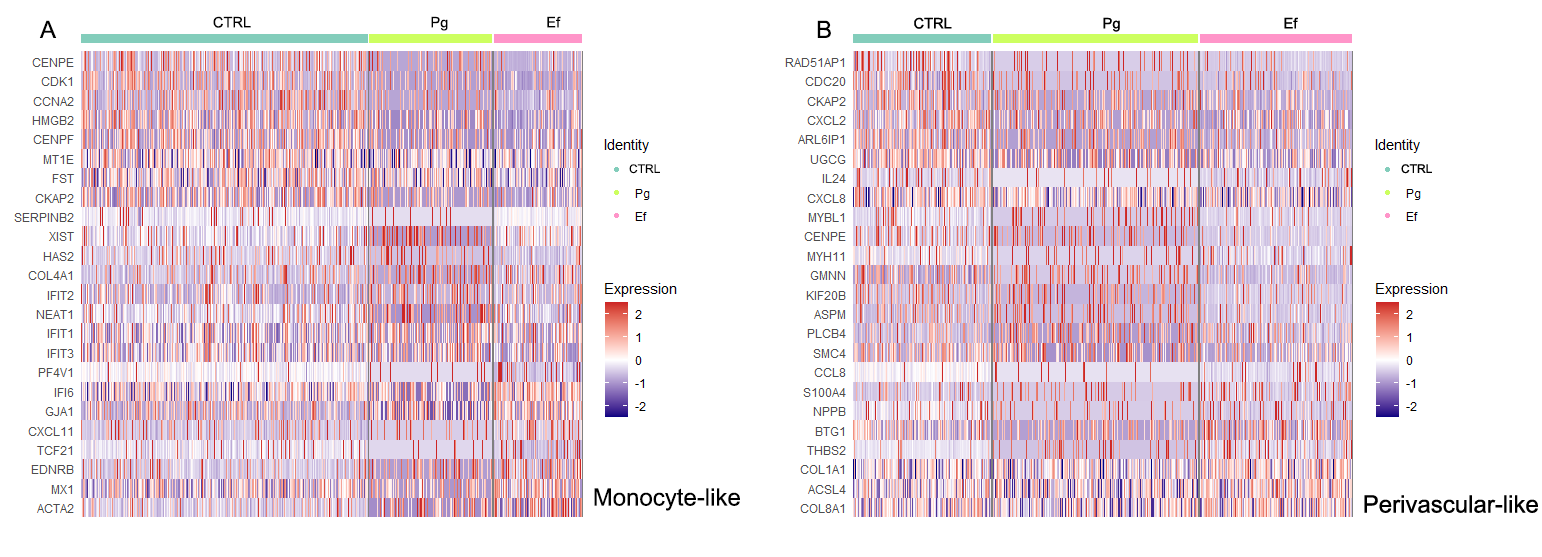

Supplement: Supplementary file 2 [file DataSheet3.ZIP › supplement figure/FigS5(A).tiff]

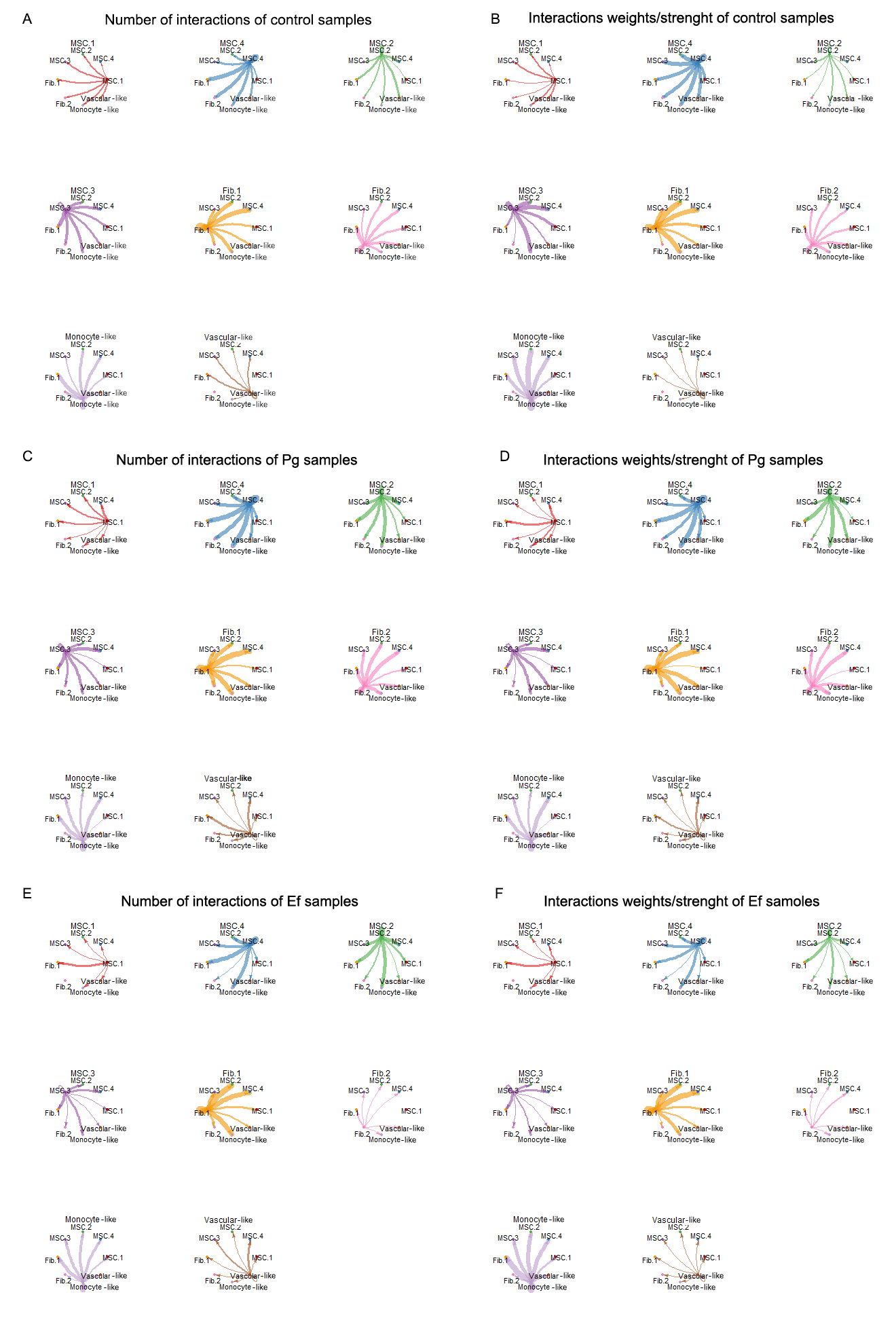

Supplement: Supplementary file 2 [file DataSheet3.ZIP › supplement figure/FigS6(A).tiff]
